# Tunable Electronic Properties of Two-Dimensional GaSe_1−*x*_Te_*x*_ Alloys

**DOI:** 10.3390/nano13050818

**Published:** 2023-02-23

**Authors:** Hsin-Yi Liu, Jhao-Ying Wu

**Affiliations:** 1 Department of Physics/QTC/Hi-GEM, National Cheng Kung University, Tainan 701, Taiwan; 2 Center of General Studies, National Kaohsiung University of Science and Technology, Kaohsiung 811, Taiwan; 3 Department of Energy and Refrigerating Air-Conditioning Engineering, National Kaohsiung University of Science and Technology, Kaohsiung 811, Taiwan

**Keywords:** nanomaterials, semiconductors, electronic properties

## Abstract

In this work, we performed a theoretical study on the electronic properties of monolayer GaSe${}_{1-x}$Te${}_{x}$ alloys using the first-principles calculations. The substitution of Se by Te results in the modification of a geometric structure, charge redistribution, and bandgap variation. These remarkable effects originate from the complex orbital hybridizations. We demonstrate that the energy bands, the spatial charge density, and the projected density of states (PDOS) of this alloy are strongly dependent on the substituted Te concentration.

## 1. Introduction

Two-dimensional (2D) monolayer graphene, with fascinating chemical and physical properties, has been extensively studied both by theoretical and experimental investigations [1,2,3]. However, being a gapless semi-metal, pristine graphene cannot be used for nano-electronic applications. This motivated several researchers to evaluate graphene derivatives, such as graphane [4], fluoro-graphene [5], metal dichalcogenides [6], oxides [7], and so on. Group III metal chalcogenides (namely MX, where M: group III element and X: S, Se, and Te) are representative 2D intrinsic semiconducting materials. The MX systems share many excellent properties of graphene, but also possess intrinsic energy gaps, and their outstanding performance in electronics and optoelectronics are well documented [8,9,10,11]. For example, InSe-based field-effect transistors exhibit ultrahigh carrier mobilities [12,13], surpassing all other semiconductor-based electronics with the same device configuration. Further, GaSe-based photodetectors display excellent optical properties, such as a fast response of 0.02 s, high responsivity of 2.8 AW${}^{-1}$, and high external quantum efficiency of 1367% at 254 nm [14]. Further, GaTe is considered as a promising material for thermoelectric devices [15], photocatalysts [16], and radiation detectors [17]. Importantly, the electron/hole density and size of the energy gap of the MX systems can be adjusted by changing the compound composition. The carrier density and band-gap engineering of these systems is crucial for obtaining a superior photoelectric power.

To date, alloying has remained one of the best approaches for the band-gap engineering of 2D semiconductors [18,19,20]. The introduction of guest atoms into a typical semiconductor can change the periodic potential and electron-ion interactions, and thus facilitate band-gap tuning. For producing such alloys, a few modern growth techniques, such as the molecular beam epitaxial (MBE) [21,22] and physical vapor transport (PVT) [23,24], have been employed. In particular, the PVT method enables the synthesis of alloys with two components containing different phases via the kinetic factor control. For example, a biphasic region with coexisting hexagonal and monoclinic phases can be created under a specific growth pressure [25,26,27]. The synthesis of complex 2D structures in the form of binary, ternary, or quaternary compounds is critical for researchers to find the practical applications of these materials.

In recent years, 2D GaSe${}_{1-x}$Te${}_{x}$ alloys have been successfully synthesized [28,29,30,31]. The Se atoms in pristine GaSe are gradually substituted by Te. The composition parameters *x* and 1−x denote the density of the added Te and the remaining Se atoms in a unit cell, respectively. The ratio of Te to Se atoms can be examined by Raman spectroscopy [32], photoluminescence spectroscopy [28], and high-resolution transmission electron microscopy [33]. Despite great experimental progress, a comprehensive theoretical study on the electronic properties of 2D GaSe${}_{1-x}$Te${}_{x}$ alloys is still lacking. In this work, we employed the first-principles density functional theory (DFT) in the Vienna ab initio Simulation Package (VASP) to systematically explore the essential properties of the monolayer GaSe${}_{1-x}$Te${}_{x}$. Detailed geometric parameters, a whole feature of energy bands, spatial charge density, and sophisticated projected density of the states (PDOSs) and their dependence on the Te-concentration are presented. The atom substitution modified the geometric structure and generated a highly heterogeneous chemical environment. The feature-rich energy dispersions include composite/oscillating parabolic subbands, many constant-energy loops, and several saddle points and partially flat bands, as well as frequent noncrossing, crossing, and anticrossing behaviors. Further, we used the spatial charge density distribution and atom- and orbital-decomposed van Hove singularities to demonstrate the complicated multi-orbital hybridizations among Ga-Ga, Ga-Se, and Ga-Te bonds. The above information is helpful in understanding the important features in the energy bands, including the nonmonotonous energy dispersions, significant π and σ band mixings, and the existence of quasi-flat and anticrossing bands.

## 2. Methods

The geometric and electronic properties of the GaSe${}_{1-x}$Te${}_{x}$ systems are investigated by the first-principles density functional calculations using the Vienna ab initio simulation package (VASP) [34,35]. The electron exchange and correlation energies are calculated by the Perdew–Burke–Ernzerhof (PBE) functional under the generalized gradient approximation [36,37]. Moreover, the projector-augmented wave PBE pseudopotentials are used to evaluate the electron-ion interactions. A vacuum distance along the z-axis is set to be 15 Å to avoid the interaction between the adjacent unit cells. The k-point mesh is set as 9×9×1 in the geometry optimization and charge density distribution, 100×100×1 in the band structure calculations, and 200×200×1 in the DOS calculations within the Gamma scheme. A plane-wave basis set with a cut-off energy of 500 eV is chosen for the valence electron wave functions. The structural relaxation is performed under a fixed cell shape and volume. All atomic coordinates were relaxed (therefore, the Ga-Ga bond is not constrained to being perpendicular to the layer) until the Hellmann–Feynman force was less than 0.01 eV/Å, along with a total energy difference of $\Delta E<10^{-5}$ eV.

## 3. Geometric Structures

**Figure 1:**
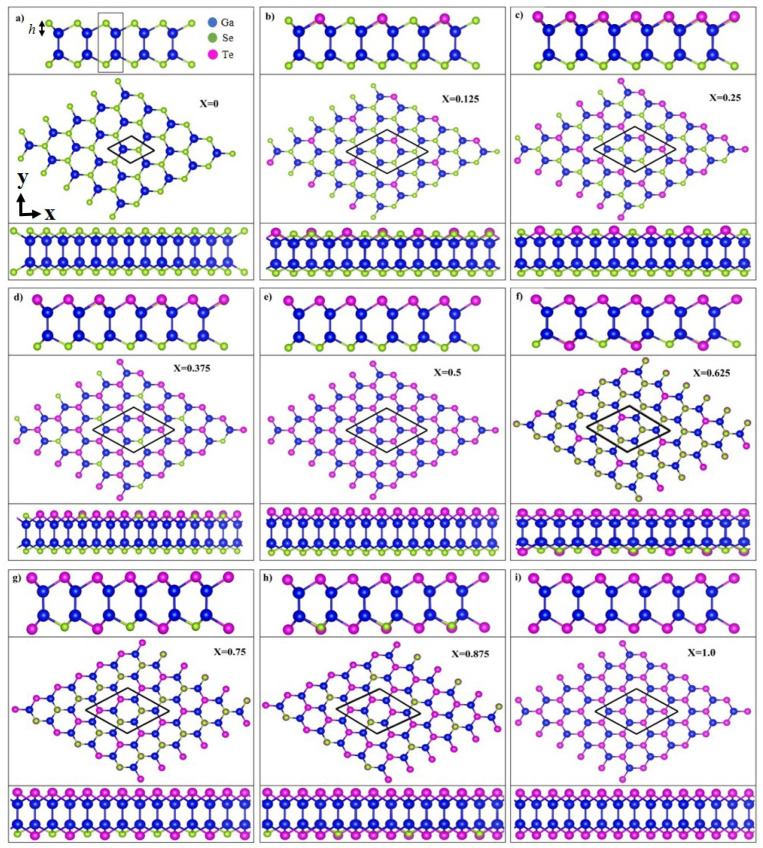
Top and two side views of the optimal geometric structures of GaSe${}_{1-x}$Te${}_{x}$ with nine different configurations x=0–1 in (**a**–**i**). The Ga, Se, and Te atoms are represented by the blue, green, and magenta balls, respectively. The buckling height *h* is indicated in (**a**).

To ensure the study is systematic, nine monolayer GaSe${}_{1-x}$Te${}_{x}$ compositions are considered, including two binary pristine systems, namely GaSe (x=0) and GaTe (x=1), and seven ternary ones (*x* = 0.125–0.875). After structural relaxation, these compositions reached their equilibrium conditions and exhibited the similar honeycomb lattice structures, as shown in Figure 1a–i. The structure consistency results in a regularly decreasing $E_{g}$ with an increase in *x* (Table 1). The middle panels, as viewed from the top, exhibit a honeycomb structure, and the upper/lower side views reveal a buckling structure along the zigzag (x-)/armchair (y-) direction. Each monolayer system comprises four atomic planes, which are all covalently bonded. The various geometric parameters are listed in Table 1, including the Ga-Ga/Ga-Se/Ga-Te bond length and buckling height *h*. The buckling height *h* is determined by the position difference along the z-axis between the nearest neighboring Se and Ga (Te and Ga) atoms, as illustrated in Figure 1a. The structural deformation after introducing the Te atoms was significant, as reflected in the large fluctuations in bond lengths and buckling heights. The Ga-Ga, Ga-Se, and Ga-Te bond lengths in the ternary alloys are 2.425–2.484, 2.451–2.483, and 2.606–2.637 Å, respectively, which are slightly larger than that of Si-Si in silicene and almost equal to that of Ge-Ge in germanene [38]. Notably, two bond lengths of Ga-Ga/Ga-Se/Ga-Te coexist in most configurations except x=0.5. In x=0.5, the Se and Te atoms are located on the opposite sides of the Ga atoms, and therefore the Ga-Ga/Ga-Se/Ga-Te bond length becomes unique. Additionally, at a low or moderate concentration, i.e., x=0.125–0.625, the buckling height is dependent on the atom site. The smaller the *x* is, the wider the range of *h* obtained. The bond length is not monotonically dependent on *x*. However, the average buckling height generally increases with theincreasing *x*. The multiple and *x*-dependent bond lengths and buckling heights are attributable to the competition between Se and Te atoms. Differences in their atom sizes and electronegativities result in nonuniform chemical environments and enrich the orbital hybridizations among the Ga-(4s, $4p_{x}$, $4p_{y}$, $4p_{z}$), Se-(4s, $4p_{x}$, $4p_{y}$, $4p_{z}$), and Te-(5s, $5p_{x}$, $5p_{y}$, $5p_{z}$) orbitals. Specifically, the increased buckling height enhances the nonorthogonality of the π and σ bondings, leading to more prominent $sp^{3}$ hybridizations. All the systems exhibit a semiconducting property. The band gap calculations with PBE and HSE06 hybrid functional are listed in the second-last and last columns in Table 1, respectively. Although a difference in band gap exists between PBE and HSE06 calculations, the trend in the band gap change is the same when the Te-concentration is increased.

## 4. Electronic Properties

### 4.1. Energy Bands

**Figure 2:**
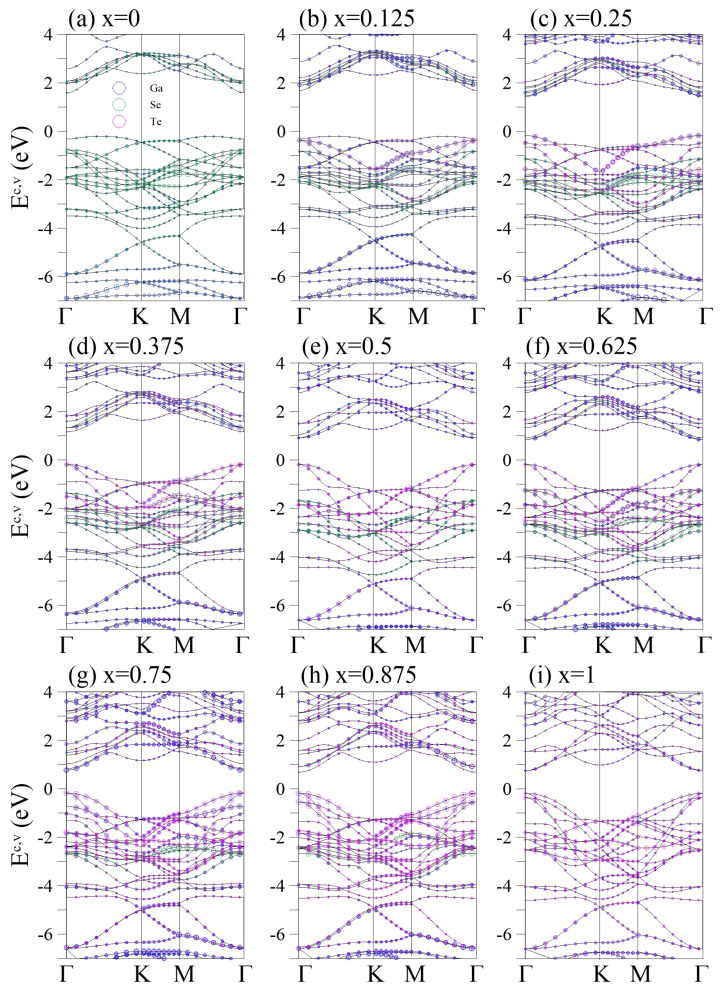
Two-dimensional band structures of the GaSe${}_{1-x}$Te${}_{x}$ alloys for different *x* in (**a**–**i**). The Ga-, Se-, and Te-atom contributions are represented by the blue, green, and magenta circles, respectively.

**Figure 3:**
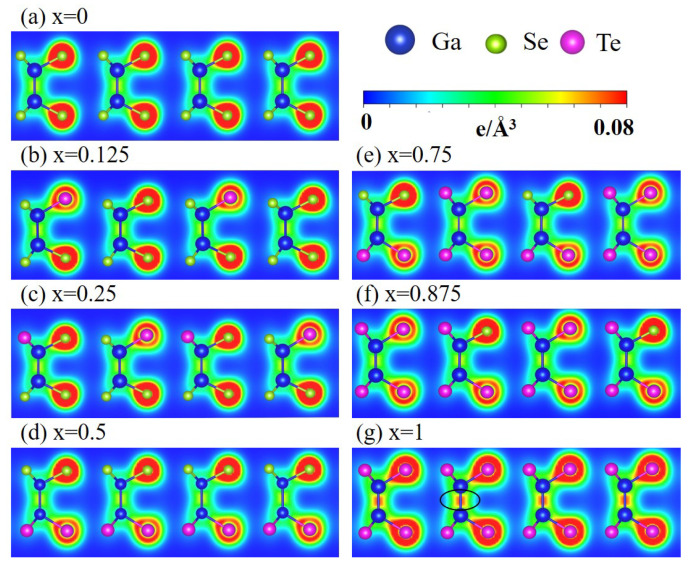
Spatial charge distributions of the GaSe${}_{1-x}$Te${}_{x}$ alloys for different *x* in (**a**–**g**). The Ga, Se, and Te atoms are represented by the blue, green, and magenta balls, respectively. The plane (1 1 0) was used to cut through the unit cell.

The electronic properties of pristine GaSe are shown in Figure 2a. The energy dispersions exhibit highly anisotropic behaviors along different high-symmetric points. The conduction bands are highly asymmetric to the valence bands about the Fermi level $E_{F}=0$. The system is an indirect-gap semiconductor, with the lowest unoccupied states being located at the Γ point and the highest occupied states lying between the Γ and K points. The latter belongs to the π band that starts at the K valley and exhibits a composite parabolic dispersion along the K →Γ→ M, with a saddle Γ-point at −1.5 eV. Such a saddle point can accumulate several electronic states and cause a logarithmically divergent van Hove singularity. In various carbon-related $sp^{2}$-bonding systems, such as layered graphene [39,40,41,42], carbon nanotubes [43,44,45], and graphite [46,47,48,49], the saddle points are responsible for the principle optical absorption peak at low frequencies. The atom-projected energy bands reveal that the contributions of Ga and Se to the low-lying π bands are almost equal. The highest occupied states of the σ bands are located at the Γ point and $E^{v}\approx -0.8$ eV. All the σ bands are distributed in the region of (−0.8, −4) eV and they anticross the *s* bands prominently around $E^{v}\approx -3.5$ eV, where the $sp^{3}$ hybridization is prominent. The energy of $sp^{3}$ hybridization is higher than that of monolayer graphene (at $E^{v}\approx -10$ eV), which is attributed to the buckled structure of the monolayer GaSe. Se (4$p_{x}$, 4$p_{y}$) orbitals predominate the σ bands, leading to an unbalanced charge distribution in the Se-Ga bond (seen in Figure 3). The VI-group element in the III-VI semiconductor determines the highest occupied π and σ states and the band gap size. Therefore, band-gap modulation can be achieved by alternating the VI-group element.

The energy bands of different substituted Te concentrations are shown in Figure 2b–i. At a low concentration (x=0.125 in Figure 2b), the system is still an indirect-gap semiconductor but $E_{g}$ increases slightly. The energy spacing between the highest occupied π and σ bands decreases owing to the increase in the energy of the Te-dominant σ band, whose ionization energy is lower than that of Se. Further, several band splittings are present, especially at (−1.6, −3.2) eV, which arise from the multiple bond lengths and buckling heights.

The highest occupied states are shifted to the Γ point when *x* is further increased to x=0.25 (Figure 2c). Accordingly, the system is transformed into a direct-gap semicondoctor, with a significant reduction in $E_{g}$. The direct $E_{g}$ is identical to the threshold optical absorption frequency and can be measured via high-resolution optical spectroscopies. The distribution range of the σ bands is extended to (−0.2, −4.3) eV, and the *s* bands are moved to the deeper energy ($E^{v}<-3.9$ eV). Further, newly created/enhanced band anticrossings and splittings are present, especially at (0, −2) eV, corresponding to the increased buckling heights and highly nonuniform chemical environments of the Te and two Se sites (see Figure 1c). For example, the two separated quasi-flat bands around −0.8 eV along the K-M path are predominated by Se and co-dominated by Te and Se.

Increasing *x* to 0.375 enlarges the σ band width and further reduces $E_{g}$ (Figure 2d). Several low-lying oscillating subbands occur at (0, −2) eV following the creation of extra band-edge states. Ga, Se, and Te codominate these low-lying bands. The anticrossing regions denote the comparable independent components of these atoms. When Te is fully substituted into one side of the system (x=0.5 in Figure 2e), the Ga-Ga/Ga-Se/Ga-Te bond length becomes unique, which enhances the band degeneracy and creates frequent band crossings. However, adding Te atoms to the other side creates new band splittings and anticrossings, as shown in Figure 1f,g for x=0.625 and x=0.75, respectively. Notably, the σ band width is almost unchanged beyond x=0.5. This may be due to the fluctuation in the crystal structure being less than those in x=0–0.5. In general, distinguishing the π and σ bands at a high Te concentration is difficult because they are fully mixed along all directions. For x=1 (Figure 2i), the intrinsic GaTe exhibits prominent anticrossings between the π and σ bands, unlike the crossing behaviors in the intrinsic GaSe (x=0). Specifically, the energy gap at x=1 ($E_{g}=0.98$ eV) is larger than that at x=0.875 ($E_{g}=0.91$ eV in Figure 2h)), which may be related to the recovering structural inversion symmetry. The large band gap variation $E_{g}=1.70\to 1.77\to 0.91\to 0.98$ eV for x=0–1 is beneficial for band-gap engineering.

The effective masses of carriers (EMC) are frequently used to interpret experimental observables for alloys. Such quantities are inversely proportional to the curvature of the electronic dispersion in a reciprocal space, implying that band edges with stronger (weaker) dispersions result in smaller (larger) effective masses. Therefore, EMCs are readily derived from the energy bands by fitting the band dispersion with a second- or third-order polynomial schematically [50,51]. The theoretical prediction of the energy dispersions can be further verified through high-resolution angle-resolved photoemission spectroscopy (ARPES). The low-energy valence bands of layered graphene systems have been identified [52], including the linear Dirac-cone structure in monolayer graphene; parabolic and linear dispersions in bilayer and trilayer AB stackings; the linear, partially flat, and Sombrero-shaped bands in trilayer ABC stacking; and the semimetallic property of bulk Bernal graphite. These diverse electronic energy spectra are consistent with the results from first-principles calculations [53]. Similar experimental measurements can be made to determine the electronic properties of GaSe${}_{1-x}$Te${}_{x}$, such as the dependence on the wave vector; large band gap; highly asymmetric electron and hole energy spectra; and frequent noncrossing, crossing, and anti-crossing behaviors.

### 4.2. Spatial Charge Density

The spatial charge density reveals the pure (orthogonal) or hybridized (nonorthogonal) π and σ chemical bonds. In Figure 3, the green parts represent the weak but significant π bondings formed by the 4$p_{z}$-4$p_{z}$ of Ga-Ga/Ga-Se and 4$p_{z}$-5$p_{z}$ of Ga-Te. The yellow and red parts represent the strong σ bondings that are mainly composed of (4$p_{x}$, 4$p_{y}$) and (5$p_{x}$, 5$p_{x}$) orbitals. The asymmetric charge distribution relative to the bond center reflects the different number of the outermost electrons and affinities of Ga, Se, and Te atoms. The charges are mostly localized around Se and Te. In general, completely separating the π and σ bondings is difficult, especially in the presence of Te atoms because of the enhanced buckled structure and $sp^{3}$ hybridization. Increasing the Te concentration results in a more prominent rectangular yellow/red part in Ga-Ga. The charge density in Ga-Te also increases due to the shortened bond length. The above features reflect the decreased/increased single/multiorbital hybridization after Te substitutions. Briefly, the competition among π, $sp^{2}$, and $sp^{3}$ bondings complicate the charge distributions and diversify the bonding strengths.

### 4.3. Density of States

**Figure 4:**
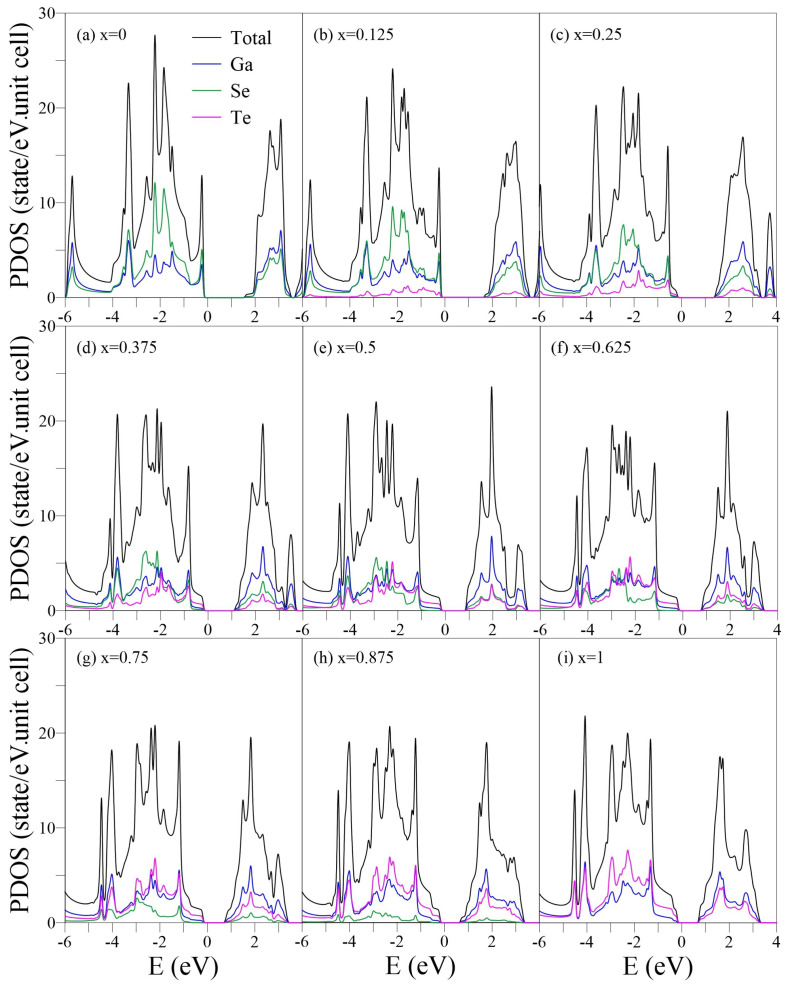
The total DOSs of the GaSe${}_{1-x}$Te${}_{x}$ alloys for x=0–1 in black curves in (**a**–**i**). The Ga, Se, and Te atom compositions are represented by the blue, green, and magenta curves, respectively.

The number, position, intensity, and composition of the van Hove singularities in the density of states (DOSs) reflect the main features of energy bands and the substitution effects. The special energy band structures, namely, the extreme states of parabolic dispersions, saddle points, and constant-energy loops can result in prominent shoulders, logarithmically symmetric peaks, and asymmetric structures in the square-root form, respectively, as shown in Figure 4. At x=0 (Figure 4a), the threshold asymmetric peak, corresponding to the band-edge states between the Γ and K points of the highest occupied parabolic π band, are codominated by Ga and Se. The shoulder structures at −0.8 and −4 eV are related to the highest and lowest valleys of pure σ bands, respectively, which determine the σ band width. Apparently, Se predominates in this region. The logarithmically symmetric peaks, which usually denote multiorbital hybridizations, could arise from the saddle points, partially flat bands, or band anticrossings. For example, the three symmetric peaks at −1.5,−2.2, and −2.5 eV correspond to the saddle Γ-points and that at −3.4 eV is associated with the partially flat bands along the Γ-K and Γ-M paths. Notably, the symmetric peak at E=−3.4 eV corresponds to the highest occupied $sp^{3}$ hybridization energy, which is higher than the lowest valley energy of the σ bands (the shoulder structure at E=−4 eV). This is opposite to that in planar monolayer graphene, in which the $sp^{3}$ peak is beyond the σ energy region. The energy overlap of $sp^{3}$ and σ results in significant band anticrossings at (−3,−4) eV, as shown in Figure 2a.

The introduction of Te atoms creates the extra constant-energy loops in the energy bands (because of the band splittings and anticrossings) and generates the additional asymmetric peaks and twin-peak structures in the DOSs (Figure 4b). The twin-peak structures (for example at E≈−1 eV or −1.8 eV) reflect the multiple chemical bonds and are composed of different weights of Se and Te atoms. The shoulder structure related to the highest σ band valley shifts towards the threshold π peak at −0.2 eV, transforming into an asymmetric peak. The transformation arises from the π-σ mixing. DOSs smaller than −2 eV are almost unchanged because of the rigid Ga-Se bonds. With x>0.125 (Figure 4c–i), the threshold DOSs exhibit a Te-dominated shoulder structure, which corresponds to the highest occupied states at the Γ point (Figure 2c–i) and is responsible for the transformation from an indirect- to direct-gap semiconductor. The other prominent peaks shift towards a lower energy with their spacings enlarged. This may be attributed to the relatively weak Ga-Te bondings. Notably, for x>0.5, the $sp^{3}$ symmetric peaks in (−4,−4.5) eV become less dependent on *x* in terms of position and intensity, exhibiting a rigid geometric structure at a sufficiently high Te concentration.

The PDOSs, which represents the decomposition of the single-/multi-orbital hybridization, are useful for distinguishing the pure and impure π/σ bonds in the crystalline phase [54,55]. Generally, a single $p_{x}$, $p_{y}$, or $p_{z}$ orbital (the Cartesian coordinates (x, y, z) are specified in Figure 1a) has σ and π components, which can be obtained by projecting the orbital along the direction parallel and perpendicular to the σ bond, respectively. In some specific cases, e.g., planar monolayer graphene in the x-y plane, the $p_{z}$ orbital has only a π component but no σ component. However, in a curved (e.g., carbon nanotube) or buckled (e.g., monolayer silicene or GaSe) structure, the $p_{z}$ orbital has both σ and π components. The curvature or buckled angle determines the ratio between the σ and π.

**Figure 5:**
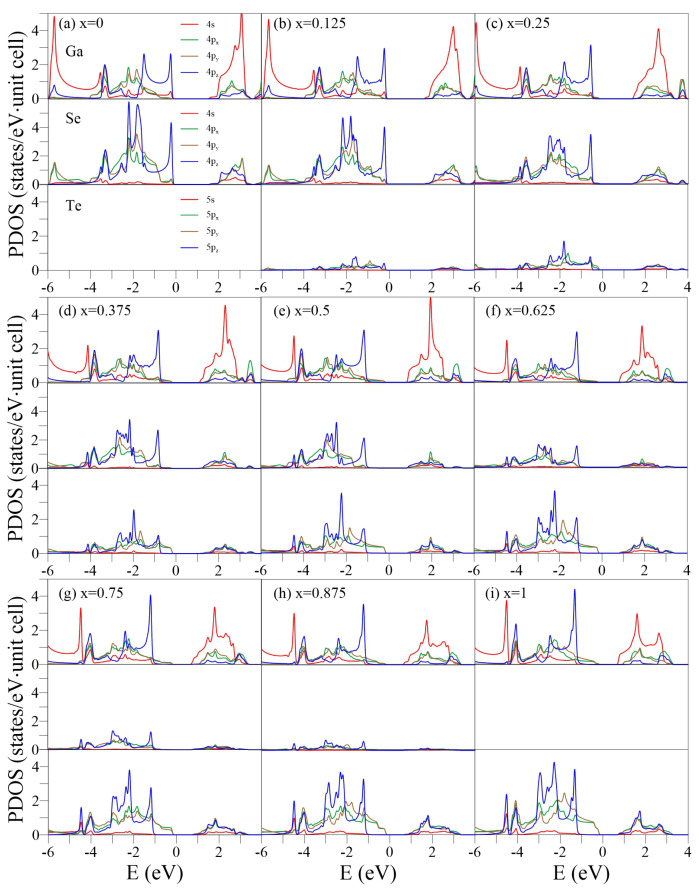
PDOSs of the GaSe${}_{1-x}$Te${}_{x}$ alloys for x=0–1 in (**a**–**i**). In each case, the up, middle, and low panels represent the Ga-, Se-, and Te-PDOSs, respectively.

For the pristine GaSe (Figure 5a), the π and σ components in (0, −2) eV can be distinguished based on the distinct structures of $p_{z}$- and $p_{x}$-/$p_{y}$-PDOSs. The 4$p_{z}$ orbitals of Ga and Se dominate the threshold DOSs due to the weak π-bondings, while the (4s, 4$p_{x}$, 4$p_{y}$) orbitals have little but significant contributions. This is in contrast to the PDOSs of monolayer graphene, in which C-(2s, 2$p_{x}$, 2$p_{y}$) orbitals are absent in the initial energy [38]. The prominent (4$p_{x}$, 4$p_{y}$, 4$p_{z}$) hybridizations of Se occur at E=−1.5, −1.9, and −2.2 eV, which correspond to the saddle Γ points, whereas the (4s, 4$p_{x}$, 4$p_{y}$, 4$p_{z}$) hybridizations of both Ga and Se appear at E=−3.4 eV, corresponding to the partially flat bands. The 4*s* orbital of Ga dominates the DOSs in E<−3.5 eV.

Increasing *x* enhances the ($p_{x}$, $p_{y}$, $p_{z}$) hybridizations of Ga, Se, and Te and induces the extra subpeaks. The shoulder structure, which is mainly composed of Te-(5$p_{x}$, 5$p_{y}$) orbitals, approaches $E_{F}$ and becomes the threshold structure of the whole DOSs at *x* = 0.25–1. The Se-PDOSs exhibit another prominent shoulder structure at a lower energy E≈−1.5 eV (the middle panel in Figure 4c) due to the stronger Ga-Se bonds. The Ga-Se/Ga-Te bonds are responsible for the indirect-/direct-semiconductor feature. In general, the different Ga, Se, and Te orbitals exhibit distinct distribution weights, suggesting that the chemical environments are highly nonuniform. The pure $p_{z}$ or $p_{x}$/$p_{y}$ contribution almost disappears beyond E=−2 eV. The several overlaps in prominent structures from different orbitals and atoms reveal the presence of complex multiorbital hybridizations, making it difficult to determine π and σ bonds or single and double bonds.

The above DOSs’ calculations can be further validated by the high-resolution scanning tunnelling spectroscopy, which is the most efficient and accurate method of examining the van Hove singularities in the DOSs. These characterization techniques have been applied to numerous well-known layered materials, such as the few-layer graphene [56], silicene [57,58], and monochalcogenides [59]. However, compared with the DOSs of graphene and silicene, those of the GaSe${}_{1-x}$Te${}_{x}$ alloys are more complex, and it is difficult to identify the bonding types at most energies. For theoretical investigations simulating the alloy systems, unlike those of graphene and silicene, using phenomenological models is almost impossible.

## 5. Conclusions

The feature-rich electronic properties of the GaSe${}_{1-x}$Te${}_{x}$ alloys were explored via first- principles calculations. The introduction of guest Te atoms modified the geometric structure, resulting in the unusual crystalline asymmetry, and diversified the electronic properties. The atom-dominated energy spectra, spatial charge density, and PDOS confirmed the coexistence of single- and multi-orbital hybridizations in the pure or impure form and demonstrated that the electronic properties were strongly dependent on the substituted Te concentration. The tunable electronic properties suggest possible applications of this unique material (alloy) to photoelectric generators, solar energy harvesting, and radiation detectors.

Finally, we would like to mention that several theoretical and experimental studies have reported various polymorphs of 2D GaSe and GaTe [60,61,62,63,64]. The different ratios of in-plane Ga-Ga and Ga-Se (Ga-Te) bonds give rise to various metastable states with similar energies. The possibility of existence of the Ga-Se-Se-Ga (Ga-Te-Te-Ga) series or Se-Ga (Te-Ga) two-atom slabs is an open issue. Although the investigation on polymorphs is beyond this work, we intend to discuss them in the future.

## Figures and Tables

**Table 1:** Geometric parameters of GaSe${}_{1-x}$Te${}_{x}$ systems: the Ga-Ga, Ga-Se, and Ga-Te bond lengths, the buckling heights *h*, the ground state energies $E_{0}$ (per unit cell), and bandgaps $E_{g}$.

Configuration	Ga-Ga	Ga-Se	Ga-Te	*h*	$E_{0}$	$E_{g}$ (PBE)	$E_{g}$ (HSE06)
Ga${\mathrm{Se}}_{1-x}{\mathrm{Te}}_{x}$	(Å)	(Å)	(Å)	(Å)	(eV)	(eV)	(eV)
x=0 (GaSe)	2.485	2.483	-	1.14	−60.842	1.70	2.67
x=0.125 (Te:Se = 1:7)	2.474; 2.484	2.469; 2.483	2.637	1.12–1.44	−59.883	1.77	2.75
x=0.25 (Te:Se = 2:6)	2.463; 2.475	2.455; 2.483	2.625	1.12–1.42	−58.857	1.58	2.41
x=0.375 (Te:Se = 3:5)	2.453; 2.465	2.454; 2.482	2.611; 2.624	1.12–1.41	−57.765	1.34	2.09
x=0.5 (Te:Se = 4:4)	2.454	2.481	2.611	1.14–1.40	−56.612	1.10	1.79
x=0.625 (Te:Se = 5:3)	2.444; 2.457	2.466; 2.481	2.608; 2.633	1.39–1.43	−55.654	1.04	1.71
x=0.75 (Te:Se = 6:2)	2.434; 2.447	2.451; 2.467	2.606; 2.621	1.41	−54.629	0.98	1.62
x=0.875 (Te:Se = 7:1)	2.425; 2.437	2.452	2.610; 2.621	1.40	−53.540	0.91	1.54
x=1 (GaTe)	2.427	-	2.608	1.39	−52.378	0.98	1.60

## Data Availability

The data presented in this study are available on request from the corresponding author.

## References

[1] Geim A.K., Novoselov K.S. (2007). The rise of graphene. Nat. Mater..

[2] Etxebarria G., Gomez-Uranga M., Barrutia J. (2012). Tendencies in scientific output on carbon nanotubes and graphene in global centers of excellence for nanotechnology. Scientometrics.

[3] Brida D., Tomadin A., Manzoni C., Kim Y.J., Lombardo A., Milana S., Nair R.R., Novoselov K.S., Ferrari A.C., Cerullo G. (2013). Ultrafast collinear scattering and carrier multiplication in graphene. Nat. Commun..

[4] Arora S.K., Youtie J., Shapira P., Gao L., Ma T. (2013). Entry strategies in an emerging technology: A pilot web-based study of graphene firms. Scientometrics.

[5] Inagaki M., Kang F. (2014). Graphene derivatives: Graphane, fluorographene, graphene oxide, graphyne and graphdiyne. J. Mater. Chem. A.

[6] Balis N., Stratakis E., Kymakis E. (2016). Graphene and transition metal dichalcogenide nanosheets as charge transport layers for solution processed solar cells. Mater. Today.

[7] Magne T.M., Vieira T.d.O., Alencar L.M.R., Junior F.F.M., Gemini-Piperni S., Carneiro S.V., Fechine L.M.U.D., Freire R.M., Golokhvast K., Metrangolo P. (2022). Graphene and its derivatives: Understanding the main chemical and medicinal chemistry roles for biomedical applications. J. Nanostruct. Chem..

[8] Kaner N.T., Wei Y., Jiang Y., Li W., Xu X., Pang K., Li X., Yang J., Jiang Y., Zhang G. (2020). Enhanced Shift Currents in Monolayer 2D GeS and SnS by Strain-Induced Band Gap Engineering. ACS Omega.

[9] Barraza-Lopez S., Kaloni T.P. (2018). Water Splits To Degrade Two-Dimensional Group-IV Monochalcogenides in Nanoseconds. ACS Cent. Sci..

[10] Guo Y., Zhou S., Bai Y., Zhao J. (2017). Oxidation Resistance of Monolayer Group-IV Monochalcogenides. ACS Appl. Mater. Interfaces.

[11] Ji Y., Dong H., Yang M., Hou T., Li Y. (2017). Monolayer germanium monochalcogenides (GeS/GeSe) as cathode catalysts in nonaqueous Li-O2 batteries. Phys. Chem. Chem. Phys..

[12] Chen Z., Sjakste J., Dong J., Taleb-Ibrahimi A., Rueff J.P., Shukla A., Peretti J., Papalazarou E., Marsi M., Perfetti L. (2020). Ultrafast dynamics of hot carriers in a quasi-two-dimensional electron gas on InSe. Proc. Natl. Acad. Sci. USA.

[13] Feng W., Zheng W., Chen X., Liu G., Hu P. (2015). Gate Modulation of Threshold Voltage Instability in Multilayer InSe Field Effect Transistors. ACS Appl. Mater. Interfaces.

[14] Hu P., Wen Z., Wang L., Tan P., Xiao K. (2012). Synthesis of Few-Layer GaSe Nanosheets for High Performance Photodetectors. ACS Nano.

[15] Bahuguna B.P., Saini L.K., Sharma R.O., Tiwari B. (2018). Hybrid functional calculations of electronic and thermoelectric properties of GaS, GaSe, and GaTe monolayers. Phys. Chem. Chem. Phys..

[16] Wang X., Wang Y., Quhe R., Tang Y., Dai X., Tang W. (2020). Designing strained C_2_N/GaTe(InTe) heterostructures for photovoltaic and photocatalytic application. J. Alloys Compd..

[17] Mandal K.C., Krishna R.M., Hayes T.C., Muzykov P.G., Das S., Sudarshan T.S., Ma S. Layered Gate Crystals for Radiation Detectors. Proceedings of the IEEE Nuclear Science Symposuim Medical Imaging Conference.

[18] Al-Kuhaili M.F., Kayani A., Durrani S.M.A., Bakhtiari I.A., Haider M.B. (2013). Band gap engineering of zinc selenide thin films through alloying with cadmium telluride. ACS Appl. Mater. Interfaces.

[19] Barthel A., Roberts J., Napari M., Frentrup M., Huq T., Kovacs A., Oliver R., Chalker P., Sajavaara T., Massabuau F. (2020). Ti Alloyed *α*-Ga_2_O_3_: Route towards Wide Band Gap Engineering. Micromachines.

[20] Berdiyorov G.R., Dixit G., Madjet M.E. (2016). Band gap engineering in penta-graphene by substitutional doping: First-principles calculations. J. Phys. Condens. Matter.

[21] Chegwidden S., Dai Z., Olmstead M.A., Ohuchi F.S. (1998). Molecular beam epitaxy and interface reactions of layered GaSe growth on sapphire (0001). J. Vac. Sci. Technol. A.

[22] Kojima N., Sato K., Yamada A., Konagai M., Takahashi K. (1994). Epitaxial Growth of GaSe Films by Molecular Beam Epitaxy on GaAs(111), (001) and (112) Substrates. Jpn. J. Appl. Phys..

[23] Clark G., Wu S., Rivera P., Finney J., Nguyen P., Cobden D.H., Xu X. (2014). Vapor-transport growth of high optical quality WSe_2_ monolayers. APL Mater..

[24] Tang L., Tan J., Nong H., Liu B., Cheng H.M. (2021). Chemical Vapor Deposition Growth of Two-Dimensional Compound Materials: Controllability, Material Quality, and Growth Mechanism. Acc. Mater. Res..

[25] Zhang L., Tang Y., Khan A.R., Hasan M.M., Wang P., Yan H., Yildirim T., Torres J.F., Neupane G.P., Zhang Y. (2020). 2D Materials and Heterostructures at Extreme Pressure. Adv. Sci..

[26] Pei S., Wang Z., Xia J. (2022). High pressure studies of 2D materials and heterostructures: A review. Mater. Des..

[27] Gao Y., Liu Y., Liu Z. (2021). Controllable growth of two-dimensional materials on noble metal substrates. iScience.

[28] Fonseca J.J., Horton M.K., Tom K., Yao J., Walukiewicz W., Dubon O.D. (2018). Structure-Property Relationship of Low-Dimensional Layered GaSe_x_Te_1-x_ Alloys. Chem. Mater..

[29] Desrat W., Moret M., Briot O., Ngo T.H., Piot B.A., Jabakhanji B., Gil B. (2018). Superconducting Ga/GaSe layers grown by van der Waals epitaxy. Mater. Res. Express.

[30] Fayek S.A. (2003). Study of non-isothermal kinetics, electrical and optical properties of (GaSeTe) films. Vacuum.

[31] Susoma J., Lahtinen J., Kim M., Riikonen J., Lipsanen H. (2017). Crystal quality of two-dimensional gallium telluride and gallium selenide using Raman fingerprint. AIP Adv..

[32] Abdullaev G., Allakhverdiev K., Babaev S., Salaev E., Tagyev M., Vodopyanov L., Golubev L. (1980). Raman scattering from GaSe_1-x_Te_x_. Solid State Commun..

[33] Dobrocka E., Vara I., Wallenberg L.R. (2001). Simulation of electron diffraction patterns from III-V alloys with CuPt ordering: Effect of clusters and antiphase boundaries. J. Appl. Phys..

[34] Kresse G., Furthmuller J. (1996). Efficient iterative schemes for ab initio total-energy calculations using a plane-wave basis set. Phys. Rev. B.

[35] Kresse G., Joubert D. (1999). From ultrasoft pseudopotentials to the projector augmented-wave method. Phys. Rev. B.

[36] Irelan R.M., Henderson T.M., Scuseria G.E. (2011). Long-range-corrected hybrids using a range-separated Perdew-Burke-Ernzerhof functional and random phase approximation correlation. J. Chem. Phys..

[37] Paier J., Hirschl R., Marsman M., Kresse G. (2005). The Perdew-Burke-Ernzerhof exchange-correlation functional applied to the G2-1 test set using a plane-wave basis set. J. Chem. Phys..

[38] Liu H., Lin S.Y., Wu J. (2020). Stacking-configuration-enriched essential properties of bilayer graphenes and silicenes. J. Chem. Phys..

[39] Lin C.Y., Wu J.Y., Ou Y.J., Chiu Y.H., Lin M.F. (2015). Magneto-electronic properties of multilayer graphenes. Phys. Chem. Chem. Phys..

[40] Do T.N., Shih P.H., Chang C.P., Lin C.Y., Lin M.F. (2016). Rich magneto-absorption spectra of AAB-stacked trilayer graphene. Phys. Chem. Chem. Phys..

[41] Koshino M. (2013). Stacking-dependent optical absorption in multilayer graphene. New J. Phys..

[42] Chiu C.W., Chen R.B. (2016). Influence of electric fields on absorption spectra of AAB-stacked trilayer graphene. Appl. Phys. Express.

[43] Shyu F.L., Lin M.F. (2002). Electronic and Optical Properties of Narrow-Gap Carbon Nanotubes. J. Phys. Soc. Jpn..

[44] Lin M.F., Shung K.W.K. (1994). Plasmons and optical properties of carbon nanotubes. Phys. Rev. B.

[45] Ruzicka B., Degiorgi L., Gaal R., Thien-Nga L., Bacsa R., Salvetat J.P., Forrio L. (2000). Optical and dc conductivity study of potassium-doped single-walled carbon nanotube films. Phys. Rev. B.

[46] Djurisic A.B., Li E.H. (1999). Optical properties of graphite. J. Appl. Phys..

[47] Taft E.A., Philipp H.R. (1965). Optical Properties of Graphite. Phys. Rev..

[48] YIchikawa H., Kobayashi K. (1966). Optical properties of graphite in the infrared region. Carbon.

[49] Dovbeshko G.I., Romanyuk V.R., Pidgirnyi D.V., Cherepanov V.V., Andreev E.O., Levin V.M., Kuzhir P.P., Kaplas T., Svirko Y.P. (2015). Optical Properties of Pyrolytic Carbon Films Versus Graphite and Graphene. Nanoscale Res. Lett..

[50] Wang V., Xu N., Liu J.-C., Tang G., Geng W.-T. (2021). VASPKIT: A user-friendly interface facilitating high-throughput computing and analysis using VASP code. Comput. Phys. Commun..

[51] Haastrup S., Strange M., Pandey M., Deilmann T., Schmidt P., Hinsche N., Gjerding M. (2018). The Computational 2D Materials Database: High-throughput modeling and discovery of atomically thin crystals. 2D Mater..

[52] Mo S.K. (2017). Angle-resolved photoemission spectroscopy for the study of two-dimensional materials. Nano Converg..

[53] Nery J.P., Mauri M.C.a.F. (2020). Long-Range Rhombohedral-Stacked Graphene through Shear. Nano Lett..

[54] Sharma P., Sundaram M.M., Watcharatharapong T., Laird D., Euchner H., Ahuja R. (2020). Zn Metal Atom Doping on the Surface Plane of One-Dimesional NiMoO_4_ Nanorods with Improved Redox Chemistry. ACS Appl. Mater. Interfaces.

[55] Sharma P., Sundaram M.M., Watcharatharapong T., Jungthawan S.H., Ahuja R. (2021). Tuning the Nanoparticle Interfacial Properties and Stability of the Core-Shell Structure in Zn-Doped NiMoO_4_@AWO_4_. ACS Appl. Mater. Interfaces.

[56] Hsu C.C., Teague M.L., Wang J.Q., Yeh N.C. (2020). Nanoscale strain engineering of giant pseudo-magnetic fields, valley polarization, and topological channels in graphene. Sci. Adv..

[57] Resta A., Leoni T., Barth C., Ranguis A., Becker C., Bruhn T., Vogt P., Lay G.L. (2013). Atomic Structures of Silicene Layers Grown on Ag(111): Scanning Tunneling Microscopy and Noncontact Atomic Force Microscopy Observations. Sci. Rep..

[58] Pham H.D., Gumbs G., Su W.P., Tran N.T.T., Lin M.F. (2020). Unusual features of nitrogen substitutions in silicene. RSC Adv..

[59] Hosseini S.A., Zad A.I., Berahman M., Mahyari F.A., Shokouh S.H.H. (2018). Scanning tunneling spectroscopy of MoS_2_ monolayer in presence of ethanol gas. Mater. Res. Express.

[60] Li X., Lin M.-W., Puretzky A.A., Idrobo J.C., Ma C. (2014). Controlled Vapor Phase Growth of Single Crystalline, Two-Dimensional GaSe Crystals with High Photoresponse. Sci. Rep..

[61] Zhao Q., Wang T., Miao Y., Ma F., Xie Y., Ma X. (2016). Thickness-induced structural phase transformation of layered gallium telluride. Phys. Chem. Chem. Phys..

[62] Li X., Li L., Wu M. (2020). Various polymorphs of group III-VI (GaSe, InSe, GaTe) monolayers with quasi-degenerate energies: Facile phase transformations, high-strain plastic deformation, and ferroelastic switching. Mater. Today Phys..

[63] Tan L., Liu Q., Ding Y., Lin X., Hu W., Cai M.-Q. (2020). Effective shape-controlled synthesis of gallium selenide nanosheets by vapor phase deposition. Nano Res..

[64] Nitta H., Yonezawa T., Fleurence A., Yamada-Takamura Y., Ozaki T. (2020). First-principles study on the stability and electronic structure of monolayer GaSe with trigonal-antiprismatic structure. Phys. Rev. B.

